# The Network between Heart and Liver from the View of Persian Medicine Versus Conventional Medicine

**DOI:** 10.31661/gmj.v12i.2557

**Published:** 2023-04-18

**Authors:** Azadeh Zarei, Mehrdad Karimi, Hossein Rezaeizadeh

**Affiliations:** ^1^ Department of Traditional Persian Medicine, School of Traditional Medicine, Tehran University of Medical Sciences, Tehran, Iran

**Keywords:** Heart Diseases, Liver Diseases, Heart Liver Axis, Network Medicine, Iranian Traditional Medicine

## Abstract

Background: Liver and heart are two important organs in the human body, whose function strongly affects other organs. On the other hand, these two main organs affect each other due to common metabolic pathways. Therefore, a disorder in any of them can lead to disease in other organs. Today, using the network medicine perspective, these complex connections can be easily mapped and discovered. From the Persian medicine viewpoint, links are formed based on the material causes of diseases, while from the conventional medicine viewpoint, they are mostly formed due to metabolites, genes, and pathways. Materials and Methods: In this article, we first investigated the recent articles of conventional medicine that examine the relationship between heart and liver in the important databases. Then, we checked out the sources of Persian medicine and finally, using the RStudio software used in network medicine for text mining, we drew the communication network centered on heart and liver and their mutual causes from the perspective of Persian Medicine. Results: Examining the network connection between the heart and the liver showed that the definitions based on Persian medicine, which are based on the material causes of diseases, are very compatible with the genes, metabolites and pathways between these two organs. Conclusion: Understanding these concepts can be helpful in detecting the co-occurrence of diseases of these two organs, as well as predicting the possible occurrence of related diseases between the heart and liver, and ultimately in better treatment.

## Introduction

Heart has been known as an endocrine organ in the body for over 40 years [1]. Various conditions and diseases such as inflammatory and infectious diseases, systemic diseases, and chronic alcoholism can have serious effects on heart and liver [2][3]. Also, as some heart diseases such as heart failure affect liver, some liver diseases such as non-alcoholic fatty liver disease (NAFLD) can also cause heart failure [4][5]. Therefore, in diseases related to any of the organs of heart or liver, the simultaneous examination of these two organs is important and can predict the patient’s condition [5][6].

For example, NAFLD [1], which includes a wide range of hepatic manifestations from steatorrhea of liver to steatohepatitis, liver cirrhosis and death [2][3][5], itself is considered as a part of the metabolic syndrome. A syndrome associated with systemic disorders including cardiac complications such as high blood pressure and vascular complications. Also, studies have shown the relationship between NAFLD, coronary heart disease and myocardial infarction.

The term "interorgan communication", also known as the multiorgan communication cascade, has recently gained attention with a focus on the communication between liver and heart.

This communication pathway is also mentioned in the literature as the cardio-hepatic axis, hepato-cardiac axis, or hepato-cardiac metabolic axis.

Important relationships in this axis are closely related to the metabolic pathways of lipids and phosphatidylcholine and the regulation of bile acids.

The cardio-hepatic axis is not a one-dimensional path and the available evidence shows that several factors can influence this path. Previous studies show that in addition to systemic disorders involving both liver and heart, several heart diseases lead to liver dysfunction [5][6].

In addition, some liver diseases affect the function of heart. The evaluation of the fluctuations of the mutual mediators confirms such communication.

On the other hand, although heart has long been recognized as an endocrine organ, the mechanisms of these connections between heart and other organs are poorly understood. One hypothesis postulates the existence of a bidirectional link between two organs that are disrupted during diseases. Furthermore, these interactions appear to be diverse, including several metabolic pathways. In addition, the cardiac endocrine function is involved in the regulation of liver inflammation and lipid metabolic pathways.

A key step in the cardio-hepatic axis is the secretion of phospholipase A2 from heart, which sends signals to liver to fuel heart. A complete understanding of this pathway can promise the development of new preventive and therapeutic strategies for heart diseases.

In Persian medicine (PM), which is also called Iranian traditional medicine (ITM), cardinal organs include the three important organs of heart, liver, and brain, and the communication between them, in addition to having serious effects on each other, also has fundamental effects on the whole body. Examining the sources of ITM shows that the scholars of this field have long believed that there are important connections between different parts of the body. They also believed that these connections between some of the organs were stronger and more important. Therefore, such organ connections, including the cardio-hepatic axis, has long been the focus of PM [7][8].

## Materials and Methods

In this narrative review study, first, some important sources of PM, including "Canon of medicine" (al-Qanoun fil-Tibb) by Avicenna (980-1037 AD) [9], (Exir Azam) by Hakim Azam Khan (1829-1902 AD), and the "Book of Treatment" (Moalejat-e-Aghili) by Mohammad Hossein Aghili Khorasani Shirazi (18th century AD), have been reviewed [7][8][10].

Then, by using text mining software including Python (version 3.8.6, USA) and RStudio (version 1.8.8., USA), the desired texts were analyzed and text mined [11][12]. Accordingly, keywords and ontologies related to heart and liver diseases and their causes were extracted. Then, using the same text mining software, especially RStudio and using packages including tm, udpipe, rebus and text2vec Subontologies were identified.

At last ggplot, coocurence, ggbipart, bipartite, and network packages of RStudio software were used to draw the network between ontologies related to diseases and their causes of two organs, On the other hand, we reviewed recent medical articles in important sources such as PubMed, Google Scholar, and Scopus, and searched them based on keywords such as heart-liver axis, liver-heart axis, liver-heart connection, cardio-hepatic interaction and hepatocardiac interaction, to extract last searches about the communication between them. Finally, we tried to summarize and compare today’s information with the data obtained from text mining of Persian medical books.

## Results

In PM, diseases are categorized according to body organs. Each of the categories include different types of diseases specific to that organ. Under each disease, after mentioning the name of the disease, the cause, the symptoms, and the treatments including pharmacotherapy with natural products, life style modifications, and/or hand manipulations (cupping, phlebotomy, leech therapy, etc.) are mentioned. According to this classification, heart diseases include 19 diseases that are divided into two categories. The first category includes heart diseases that rapidly affects the function of other organs

On the other hand, the second category of heart diseases only involves heart; however, if left untreated, it can cause general weakness, fatigue, and thinness.

Also, liver disorders include 17 diseases in PM. These diseases are also divided into two general categories in liver. The first category includes disorders directly involve liver, such as various dystemperaments [13]; whereas the second category is caused secondary to another organ disease, such as stomach, intestines, heart, brain, and kidneys. Although the stomach can cause disease in liver more than other organs due to its participation in digestive processes with liver, the effects of other organs such as heart, cannot be ignored [14, 15]. In view of PM, cardio-hepatic link is due to several mutual contributors. These connections occur either due to the mutual causes, or the cardiac complications of liver diseases and vice versa.

According to PM, the main causes of diseases in the body are categorized into four groups. One of these groups encompasses “material causes” which induce diseases by interfering with four humors: phlegm, blood, yellow bile, and black bile. From the view of PM, diseases are caused by a variety of material causes. Also, each type of material causes can be effective in the occurrence of certain categories of diseases in different organs. The relationship between the disorders and humors’ imbalance with the body’s proteomes has been described in recent studies [16]. Some types of material causes have greater effects on specific organs, which can be explained by the communications of these organs with each other.

Accordingly, in addition to the mechanisms mentioned PM textbooks, delving into the causes of heart and liver diseases would deepen our understanding of the cardio-hepatic axis.

Each of these four humors, which are known as material causes of PM, is divided into subclasses based on the changes that may occur in them. According to this classification, more than 1400 unique material causes have been identified. Of these, 87 material causes are involved in heart diseases, as well as 97 material causes in liver diseases. Also, the investigations revealed that out of the total of 184 material causes mentioned in heart and liver diseases, there are 55 mutual material causes which may result in simultaneous dysfunction of both liver and heart.

**Table:** **Table-1.** The Common Material Causes of Liver and Heart Diseases

**Material cause**	**Heart disease**	**Liver disease**
**Moisture**	Palpitation	Ascites/ Liver stone/ Liver swelling
**Wind**	Palpitations/ syncope	Ascites/ Inflammation of Liver
**Vapor**	Palpitations/ syncope/ Inflammation of Heart	Jaundice/ Liver swelling/ Inflammation of Liver
**Phlegm**	Palpitations/ syncope/ Inflammation of Heart	Ascites/ Jaundice/ Liver obstruction/ Diarrhea
**Blood**	Palpitations/ syncope/ Heart conflict	Ascites/ Jaundice/ Liver weakness
**Yellow bile**	Palpitations/ syncope/ Heart conflict	Ascites/ jaundice/ liver stone/ liver swelling
**Black bile**	palpitations/ syncope/ Heart conflict	Jaundice/ liver swelling

**Table:** Table **2.** Contribution of Heart and Liver in PM [7][17][18][19]

**Type of contribution**	**Mechanism**
**Quantitative blood change**	Liver failure leads to inadequate blood production and consequently poor nutritional supply of Heart and, consequently, cardiac failure.
**Qualitative blood change**	Excessive hepatic heat (high metabolism) causes palpitation by creating poor quality blood (choleric dystemperament).
**Qualitative blood change**	Excessive cold in Liver (low metabolism) causes weakness (Heart failure) by producing poor quality blood (phlegmatic dystemperament).
**Membrane-based connection**	Hepatic edema causes cardiac dysfunction via membrane connections.
**Blood and energy distribution**	Improper blood and energy distribution causes cardiac failure.
**Anatomical change**	Bilateral vascular connections transmit the damage of one organ to the other.

In this regard, the superclass of material causes that cause disease in these two organs, along with the corresponding disorders, is shown in Table-1, and the mechanisms proposed communication mechanisms in view of PM are shown in Table-2. Today, the conventional medical view of the cardio-hepatic axis is mostly explained based on metabolites and pathways. Observations show that liver disorders are common in heart failure and any type of right ventricular dysfunction [20].

These co-occurrences have led scientists to make detailed investigations in the field of genes, metabolites, and possible pathways. Research shows that hepatic microRNAs as non-coding RNAs can be distributed from liver into the bloodstream and affect gene expression and metabolic pathways (e.g., lipid metabolism, insulin biogenesis, and cholesterol balance) in other organs, such as heart. For example, suppression of miR-34 has been proposed as a novel therapeutic strategy against NAFLD and cardiac dysfunction, and miR-144 has been reported as a critical risk factor for cardiovascular diseases.

Also, according to today’s theories, both heart and liver are considered a endocrine organs. Studies based on this theory show that the cardio-hepatic axis affects the inflammatory process, lipid metabolism, and gene expression in liver. Additionally, it leads to an increase in triglycerides and very low-density lipoproteins and sends a message of signal energy sufficiency from heart to liver. A summary of recent findings on the cardio-hepatic axis of the endocrine system is shown in Table-3.

## Discussion

**Table:** **Table-3.** Recent Findings on the Cardio-Hepatic Axis of the Endocrine System

**Type of communication**	**Mechanism and evidence**
**Systemic conditions involving both Heart and Liver **	Inflammation, infection, chronic alcoholism, systemic diseases (e.g., metabolic diseases such as Wilson and hemochromatosis, insulin-dependent diabetes, etc.), viral hepatitis, Alagille syndrome, cytomegalovirus infection, and pulmonary diseases (e.g., obstructive sleep apnea and chronic obstructive pulmonary disease) [4][21]
**Effects of Hepcidin on Heart **	Hepcidin is a liver-derived protein with cardio protective roles [22]
**Effects of phospholipase A2 on Heart **	Phospholipase A2 is produced by Liver and prevents Heart disorders by controlling inflammation and regulating lipid metabolism [4][23]
**Effects of liver failure on cardiac function **	A decrease in the levels of plasma proteins, reduced energy supplied to Heart, and increased levels of inflammatory cytokines following hepatic insufficiency may lead to cardiac failure [22][24]
**Protective effects of Liver on Heart **	Mediated by the secretion of hepatocytes and the STAT3 pathway [25]
**Liver disorder and arrhythmia **	The higher risk of atrial fibrillation in individuals with high transaminase levels, the strong association between non-alcoholic arrhythmia and hepatitis [26][27]
**Atherosclerosis induced by hepatic dysfunction **	Inflammation, as the primary cause of atherosclerosis, is triggered by impaired lipoprotein metabolism [28]
**Effects of fatty liver disease on cardiovascular disorders **	enhances the risk of cardiovascular diseases [18]. Hepatic fibrosis is associated with cardiomyopathy and cardiovascular disorders [29][30]
**Cardiac conditions affecting Liver **	Compressive pericarditis, Corpulmonale, ischemic cardiomyopathy, mitral stenosis, pulmonary embolism, hypertension, and tricuspid insufficiency [31]
**Regulating gluconeogenesis and lipolysis in Liver **	Mediated by Ach secreted from cardiac sympathetic nerves [23]
**Protecting hepatocytes against ischemia **	Suppressing Kupffer cells during inflammation [4]
**Hepatopathy induced by Heart failure **	Cardiokine-induced hepatic cholestatic damage [31][32][33][34]
**Hepatic congestion induced by insufficiency of Hearts’ right side **	Hepatitis is caused by passive congestion and acute hepatocellular necrosis following perfusion [35]
**Effect of Heart on Liver to regulate thermogenesis and energy distribution **	Mediated by Ach secreted from cardiac cells [23]
**Protective effect of Heart on Liver **	Heart regulates lipolysis in adipocytes by secreting ANP [36]
**Bilateral cardio-hepatic route **	The myosin (R403Q) mutation decreases lipid uptake by Heart, thereby causing cardiac dysfunction and increased plasma lipid and hepatic fat storage, followed by the activation of gluconeogenesis and elevation of blood sugar, and finally, cardiovascular diseases [6]

**Figure-1:**
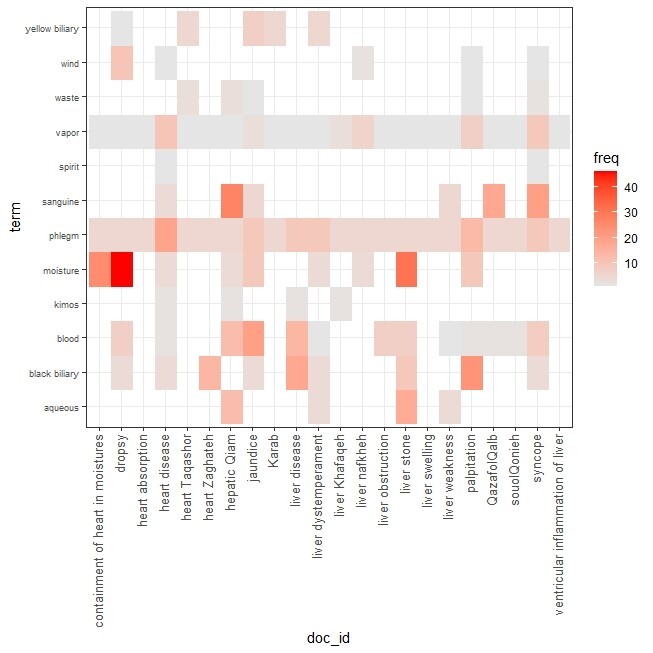
Figure1. The relationship between material causes and heart and liver diseases. The color ranges from pale to bold shows the co-occurrence of each material cause with heart and liver diseases.

PM is a holistic natural network considering different organs of the body as a whole. In other words, instead of the mere focus on one organ, mutual interactions among different organs are examined as participation that has an anatomical or histological form in some cases; while they may be physiological and functional in some other cases [37]. The relationship between liver and heart is one of the most well-recognized interorgan interactions in PM, in which liver is assumed as the central source for blood formation and heart as the blood and energy distributor in the body. Accordingly, most of the body organs interact with these two organs [7, 8]. Besides the two organs’ well-known contribution to blood production and energy intake and distribution, there also have other specialized communications, as described in Table-2.

From the perspective of PM, the role of liver in blood formation is consistent with the involvement of this organ in the production of many plasma components, mostly hormones, precursors, and lymph, and its effects on precursors, as noted for the cardio-hepatic axis [38][39]. The products of these organs in developing diseases are consistent with their roles as the cardinal organs and their involvement in blood and energy distribution. The holistic and personalized perspectives are different, and PM also considers diseases from these perspectives within a general framework. Regarding cardiac diseases, they are classified into two specific and general categories, with the latter category being consistent with the systemic effects of heart diseases [40][41]. The highest consistency in humor types is for hepatic diseases sharing similarities with cardiac abnormalities such as phlegm associated with lethargy, and consistent with heart insufficiency [19].

Membrane communications (mentioned in Table-2) indicate a mechanical viewpoint, and blood circulation participation shows hormonal and endocrine communications, as recognized by the holistic view adopted by PM scientists.

Despite the personalized viewpoint, while maintaining holism, PM adopts the same approach to the pathology, symptoms, and etiology of diseases, as well as anatomical and functional communications, thereby explaining the cardio-hepatic axis in detail.

Using network medicine software and considering PM viewpoints, important and effective connections between the causes of diseases and various diseases can be discovered. For this purpose, with a network-based medical perspective, we examined the asymmetric relationships between the discrete concepts found in PM textbooks between liver and heart disease. For this purpose, three separate ontologies were first created by using RStudio text mining software and extracting keywords related to diseases and body organs, as well as material causes mentioned in the sources of PM. These ontologies include all the specific keywords in the category. The preparation of

**Figure-2:**
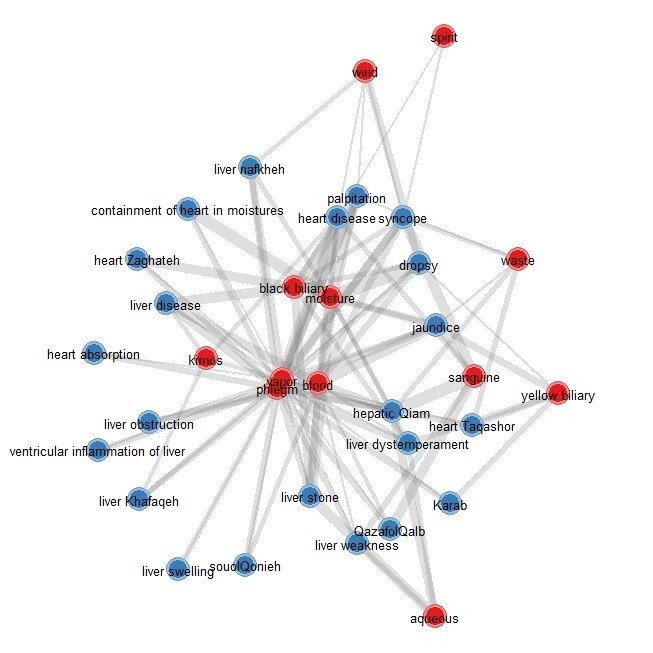
Figure-2. The relationship between Heart and liver diseases and the material causes from the perspective of Persian medicine. The blue nodes represent the disease and the red nodes show the cause of the disease. In this type of network illustration, the nodes that are in the center are the most common between diseases and the nodes that are far from the center are less common.

these ontology trees, in addition to correctly displaying the categories of each group, can also reveal the hidden connections between each ontology and other ontologies. The goal was to show the connections that have been discovered based on today’s research and based on pathways, metabolites, and genes between body organs, from the perspective of PM and based on the causes proposed for diseases.

The study of network connections between heart and liver diseases showed that the most mutual material causes that leads to disease in these two organs, is a group of material causes that cause cold and wetness in these organs. This category can be mentioned due to the material of phlegm and excess moisture in the organs.

In the next rank, the material cause of vapor can be effective in causing disease in these two organs. These connections are shown in the Figure-1 and -2. Knowing the mutual causes of diseases can in the next step predict the co-occurrence of each group of diseases with other diseases of the body. According to this point of view and using text mining and network drawing software, these connections can be illustrated. In this direction and according to this point of view, we drew the co-occurrence of diseases related to body organs with heart and liver diseases.

**Figure-3:**
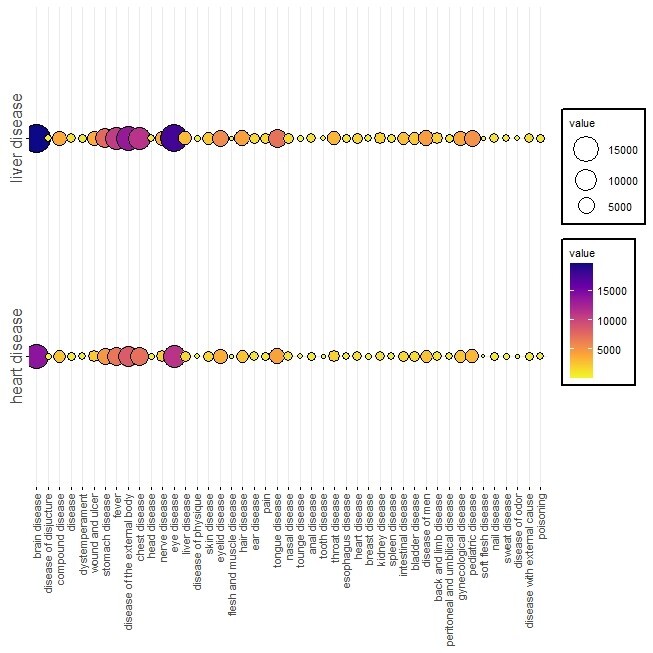
Figure-3. Co-occurrence of Heart and liver diseases with other diseases.

The interesting result was that due to the many commonalities between these two organs, the co-occurring diseases with these two organs had many similarities with each other. Based on this, the most co-occurrence of these two diseases was with brain diseases. This data is important because from the PM viewpoint, the three organs of heart, liver and brain are known as the cardinal organs, which affect each other and the whole body. In the next step, the highest co-occurrence was with eye diseases, then with external body diseases and then with fevers. This information can be of great help in determining the complications of diseases and their prognosis by predicting possible conditions. These relationships are shown in Figure-3.

**Figure-4:**
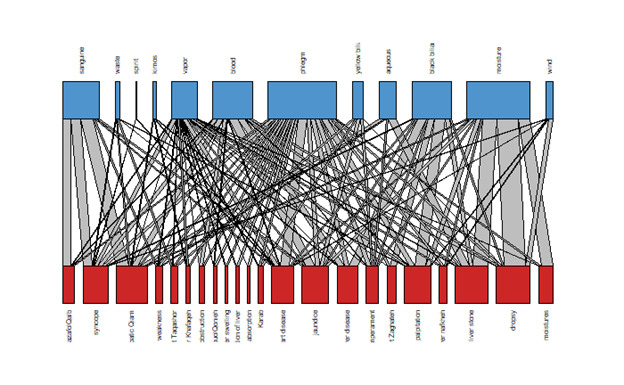
Figure-4. Bipartite network between mutual material causes and diseases of heart and liver.

These connections have also been proven today, in the form of metabolic connections. The visualization of connections between different organs of the body, which was based on network medicine, in addition to being confirmed by new researches, can raise new ideas and theories about connections between organs. Based on this, we have drawn the findings of the relationship between heart and liver from the perspective of PM and based on network medicine. Construction of the diseasome bipartite network. A bipartite disease diagram showing the link between heart and liver diseases with material causes. Stronger connections are depicted with thicker edges. This bipartite diagram is shown in Figure-4.

## Conclusion

PM is an ancient traditional medicine system with a holistic approach compared with allopathic medicine with a focus on reductionism that can be enhanced using modern systems medicine approaches. PM scholars believed that body organs are all related to each other via direct and indirect axes and the dysfunction of any organ negatively impacts its interconnected organs. One of the most important axes is a reciprocal connection between heart and liver.

Everything we know today as physiological or pathological processes and the connections and logical concepts in these processes are possible by analyzing complex networks using new software. The more the PM doctrine is related to modern medicine, the more its concepts are organized and standardized with modern science. In PM, four Aristotelian causes for diseases are stated amongst which material causes are the most important from the PM viewpoint. Knowing all the material causes mentioned in medical books and understanding the type of their effect on diseases helps a lot in treatment. A deep understanding of the terms and mechanisms of PM, as well as the discovery of complex network connections between causes and diseases from the perspective of PM, along with modern medicine can help in recognizing and explaining the causes of various diseases in the body. Different methods are needed, in the process of treatment and discovery of new drugs, with

regard to the biological activities of drugs, to give special help to the development of doctrines of traditional and complementary medicine such as PM. Future studies are essential to further clarify the interorgan connections and the related modern evidence for a better understanding of disease pathologies in traditional medical doctrines.

## Acknowledgments

The authors would like to thank Dr. Mahdi Mirzaei and Dr. Mohieddin Jafari for their valuable comments and help.

## Conflict of Interest

The authors confirm that they do not have any conflicts of interest.
